# Increased expression of chaperone proteins in response to DENV 2 infection of Huh-7 liver cells

**DOI:** 10.1371/journal.pone.0329783

**Published:** 2025-08-01

**Authors:** Chanida Chumchanchira, Wannapa Sornjai, Sittiruk Roytrakul, Pathrapol Lithanatudom, Duncan R. Smith

**Affiliations:** 1 PhD Degree Program in Biology, Faculty of Science, Chiang Mai University, Chiang Mai, Thailand; 2 Institute of Molecular Biosciences, Mahidol University, Nakhon Pathom, Thailand; 3 National Center for Genetic Engineering and Biotechnology (BIOTEC), National Science and Technology Development Agency, Pathum Thani, Thailand; 4 Department of Biology, Faculty of Science, Chiang Mai University, Chiang Mai, Thailand; Instituto Nacional de Salud Publica, MEXICO

## Abstract

The mosquito transmitted dengue virus (DENV; family *Flaviviridae*, genus *Orthoflaviviru*s, species *Orthoflavivirus denguei*) is a significant public health problem in many tropical and subtropical countries around the world. Human infection by DENV is predominantly asymptomatic in 80% of cases, but the remaining 20% of infections can result in symptoms ranging from a mild undifferentiated fever to life threatening dengue hemorrhagic and dengue shock syndrome. During infection DENV induces changes in the host cell, including changing protein expression, altering the cellular lipids and inducing changes in membrane architecture. A number of cell types have been shown to be permissive for DENV replication, including hepatocytes. This study sought to investigate the protein expression changes induced by DENV infection of a liver cell line, Huh-7, using 2-dimensional (2D) electrophoresis. At 48 hours post infection 14 protein spots were found to have altered expression as compared to mock infected cells at the same time point. In particular four of the proteins showing alterations of expression were chaperone proteins (Stress-70 protein, Endoplasmic reticulum chaperone BiP (GRP78), Heat shock 70 kDa protein 4 and Heat shock protein HSP 90-beta), of which three were upregulated (Stress-70 protein, Endoplasmic reticulum chaperone BiP (GRP78), Heat shock 70 kDa protein 4) and one was downregulated (Heat shock protein HSP 90-beta). GRP78 showed the largest change in expression amongst these four proteins, and so its expression was confirmed by western blot analysis. GRP78 has been shown by many studies to be critically involved in the replication of orthoflaviviruses, and this study further underlines the importance of this protein.

## Introduction

Dengue virus (DENV) is a mosquito transmitted virus that belongs to the family *Flaviviridae*, genus *Orthoflaviviru*s and species *Orthoflavivirus dengue* [1]. DENV is primarily transmitted to humans by female *Aedes (Ae.) aegypti* and *Ae. albopictus* mosquitoes. Studies have suggested that approximately 3.6 billion people are living in areas at risk for DENV infection and it is believed that over 400 million human infections occur each year [2], with some 22,000 deaths [3]. DENV infection of humans is asymptomatic in the majority of cases but infection can result in clinical symptoms, ranging from a mild flu-like syndrome known as dengue fever to the more severe presentations associated with hemorrhage termed dengue hemorrhagic fever (DHF) and dengue shock syndrome (DSS) [4]. To date, no specific treatment for treating DENV infection has been approved. DENV is a positive-sense single strand RNA virus and the species *Orthoflavivirus denguei* consists of four distinct viruses termed DENV 1 to DENV 4, that share some 65% of their genome sequence. The viral genome encodes a polypeptide which span 3,400 amino acids in length and is divided into 3 structural proteins (capsid, precursor of membrane and envelope) and 7 non-structural proteins; NS1, NS2A, NS2B, NS3, NS4A, NS4B and NS5 [5]. The replication of dengue virus takes place in target host cells at the endoplasmic reticulum and the immature virions are trafficked through the trans-Golgi network where maturation occurs, with subsequent release of the newly made infections virus [6]. During viral replication in the host cell DENV induces significant changes in protein expression to create an environment that favors DENV replication and dampens the host cell innate immune response [7].

The liver (including hepatocytes and Kupffer cells) is a well characterized target tissue of DENV infection [8]. Levels of the liver enzymes alanine aminotransferase and aspartate aminotransferase (indicative of liver damage) are frequently elevated in DENV patients [9], and there is an increased rates of liver failure in severe DENV patients [10]. Infected hepatocytes have been detected in autopsy specimens from people who died from DENV infection [11] and primary human hepatocytes are both susceptible and permissive to DENV [12]. Despite the importance of liver involvement in DENV infection, few studies have investigated the changes in protein expression of these cells in response to DENV infection. This study sought to investigate the changes in protein expression in Huh-7 cells in response to DENV infection, as understanding the molecular alterations in DENV infected liver cells may develop lines of evidence to either protect liver cells from the more severe consequences of DENV infection, or lead to new therapeutic targets against DENV infection.

## Materials and methods

### Cells and viruses

Human hepatocarcinoma Huh-7 cells [13] were cultured in Dulbecco’s minimal essential medium (DMEM, Gibco, Invitrogen, Grand Island, NY) and incubated at 37°C with 5% CO_2_. DENV 2 (strain 16681, NCBI Accession number NC_001474) was propagated in C6/36 (*Ae. albopictus*) cells (ATCC CRL-1660) as previously described [14]. Virus titer was determined by plaque assay on LLC-MK_2_ (Rhesus monkey kidney) cells (ATCC CCL-7).

### Plaque assay

LLC-MK_2_ were seeded in six-well plates and cultured under standard growth conditions for 24 hours. The culture medium was removed and the 10-fold serial dilution of DENV 2 virus in BA-1 (1X medium 199/Earle’s balanced salts, 0.05M Tris-HCl pH 7.6), 1% serum albumin, and 0.075% NaHCO_3_, and 100U of penicillin-streptomycin per mL) were added in each well plate and incubated at 37°C for 2 hours. Eventually, the virus dilution was removed and overlaid with 1X nutrient solution with 0.8% (W/V) SeaKem LE agarose (Merk KGaA, Darmstadt, Germany), then the infected cells were further incubated at 37°C with 5% CO_2_ for 5 days. On day 6 post infection, the infected cells were overlaid with a nutrient agarose containing 0.06% neutral red to visualize the plaques. After overnight incubation, the plaques were counted, and the viral titer was calculated.

### Virus infection

Huh-7 cells were seeded in six-well plates and cultured under standard growth conditions for 24 hours. The culture medium was removed after cells reached 80% confluency and cells were then either mock infected or infected with DENV 2 at a multiplicity of infection (MOI) of 5 for 2 hours. Subsequently, the virus containing medium was replaced by fresh culture medium and cells were further incubated under standard condition for 48 hours. All experiments were undertaken as three independent biological replicates.

### Two-dimensional (2D)-gel electrophoresis

Cell pellets from mock and DENV 2 infected Huh-7 cells were lysed using RIPA buffer (1% NP-40, 0.5% sodium deoxycholate, 0.1% sodium dodecyl sulfate, 137 mM sodium chloride, 2.7 mM potassium chloride, 4.3 mM disodium hydrogen phosphate, 1.4 mM potassium dihydrogen phosphate) containing a protein inhibitor cocktail and the proteins were precipitated overnight by the addition of acetone and methanol. Subsequently the protein pellets were dissolved in lysis C buffer (8M urea, 2M thiourea, 4% CHAPS, 20mM DTT, 1mM PMSF, 1mM benzamide) prior to determining the protein concentration by the Bradford assay [15]. Subsequently, 250 mg of purified proteins were subjected to 2D analysis exactly as described previously [16,17]. Full experimental details are given in the supplementary file, reproduced under a creative commons license from one of our prior publications [17].

### Tryptic in gel digestion and protein identification by LC-MS-MS

Differentially expressed protein spots identified by image analysis were cut from the gels and subjected to in-gel tryptic digestion essentially according to the method described in our previous study [18]. Peptide mixtures were analyzed by ultra-performance liquid chromatography (Ultimate 3000, Dionex, Sunnyvale, CA) coupled to a micrOTOF-Q II™ ESI-Qq-TOF mass spectrometer (Bruker, Billerica, MA). The MS/MS spectra produced from each sample were searched against the NCBI database using the MASCOT search engine (Matrix Science, London, United Kingdom). Full experimental details are given in the supplementary file, reproduced under a creative commons license from one of our prior publications [18].

### Western blot assay

Proteins from mock or DENV 2 infected Huh-7 cells were separated by 10% SDS-PAGE and proteins were subsequently transferred to nitrocellulose membranes. The protein containing membranes were probed with primary antibodies directed against glucose regulatory protein 78 (GRP78), DENV E protein, DENV NS1 protein and actin followed by appropriate HRP-conjugated secondary antibodies. Antibodies and dilutions used can be found in Supplemental Table S1 in S1File.

### Ontological analysis

The identified protein data was submitted to the online DAVID software functional annotation tool available at http://www.davidbioinformatics.nih.gov, and an enrichment score is calculated by comparing the proportion of the genes in 14 differentially identified protein set that are associated with a specific category to the proportion in the homo sapiens genome. The results were indicated with p < 0.05. The STRING database tool available on https://string-db.org/ version 11 was used to analyze protein-protein interaction networks. The PPI enrichment value was indicated with p < 0.05.

### Statistical analysis

The data in these experiments were considered as normally distributed and they were measured in ratio scales, therefore parametric tests, such as t-test and one-way ANOVA, were selected and conducted. All statistical analyses of the numerical data were performed using Student T-test and one-way ANOVA on GraphPad Prism version 7.0.0 (GraphPad Software, CA, USA). *P* values of less than 0.05 were considered statistically significant.

## Results

### 2D-gel analysis of differentially expressed host proteins in Huh-7 cells after DENV 2 infection

To confirm the susceptibility of Huh-7 cells to DENV infection, Huh-7 cells were either mock infected or infected with DENV 2 at multiplicity of infection (MOI) of 1, 5, 10 and 20, and the level of infection determined by flow cytometry. The results (Supplemental Fig S1 in S1File) showed that all MOI tested gave robust levels of infection on days 2 and 3 post-infection (p.i.), and an MOI of 5 was selected for further experimentation.

**Fig 1:**
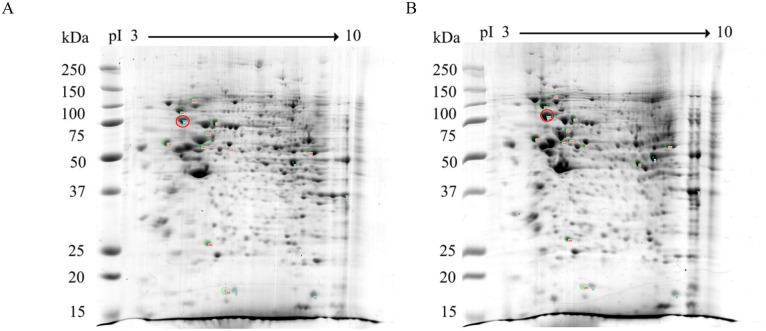
2D-PAGE analysis huh-7 cells mock infected or infected with DENV 2. Huh-7 cells were either (A) mock infected or (B) infected with DENV 2 (strain 16681) and at 48 h post infection cells were harvested and proteins extracted and separated by 2D-PAGE. Spot intensities were determined by image analysis and those showing a statistically significant difference in expression were selected for further analysis. GRP78 is circled in red.

To investigate the effects of DENV 2 infection on host protein expression, Huh-7 cells were either mock infected or infected with DENV 2 at MOI 5 independently in triplicate. At 48h post-infection cells were collected and proteins were extracted and quantitated. A time point of 48 h.p.i was selected as this precedes the onset of cytopathic effects that might serve to complicate the analysis. For proteomic analysis, protein samples were subjected to 2D-gel electrophoresis followed by staining with Coomassie Blue G250 (Fig 1). The three replicate gels are presented in Supplemental Figs S2 and S3 in S1File. A total of 14 significantly differentially expressed protein spots were identified, which were cut from the gel and after in-gel tryptic digestion the peptides were subjected to analysis by LC-MS-MS. All 14 differentially expressed spots were successfully identified (Table 1). The numbering of the spots was assigned by the 2D gel analysis program, and the Table lists them in this order. All 14 proteins were identified in all three replicates, except for two proteins in mock infection (mitochondrial Elongation factor Tu and mitochondrial glutathione reductase) which were only found in one of the three replicates- suggesting that these proteins are significantly upregulated by DENV infection (see Suppelemntal Table S2 in S1File).

**Table 1:** List of 14 proteins which are differentially expressed in Huh-7 cells after infection with DENV 2 for 48 hours.

Spot No.	Protein name	pI	Uniprot Accession	Score	Intensity			
					Mock infection	DENV-2 infection	Fold change	P value
57	Malate dehydrogenase, mitochondrial	8.92	P40926	259	601.34	2185.41	3.63	9.82E-03
84	Elongation factor Tu, mitochondrial	7.26	P49411	90	3085.81	588.37	0.19	3.64E-04
104	Heterogeneous nuclear ribonucleoprotein H	5.89	P31943	104	2384.35	2040.52	0.86	4.72E-02
129	Peptidyl-prolyl cis-trans isomerase FKBP4	5.35	Q02790	93	1011.03	1286.67	1.27	1.50E-02
136	Protein disulfide-isomerase	4.76	P07237	599	1485.14	1726.10	1.16	4.99E-02
152	Stress-70 protein, mitochondrial	5.87	P38646	712	947.03	1123.20	1.19	2.64E-02
259	Endoplasmic reticulum chaperone BiP	5.07	P11021	1191	1397.79	2219.80	1.59	1.53E-02
266	Superoxide dismutase [Cu-Zn]	5.70	P00441	75	489.35	1408.66	2.88	3.73E-02
330	Glutathione reductase, mitochondrial	8.74	P00390	124	6956.52	3122.39	0.45	3.79E-02
361	Heterogeneous nuclear ribonucleoprotein K	5.39	P61978	208	447.60	2059.36	4.60	1.00E-02
363	Heat shock 70 kDa protein 4	5.11	P34932	153	2172.94	3220.00	1.48	4.47E-02
364	Tubulin alpha-1A chain	4.94	Q71U36	427	4369.59	1126.72	0.26	3.42E-02
366	Ubiquitin carboxyl-terminal hydrolase isozyme L1	5.33	P09936	134	1013.71	1058.22	1.04	6.89E-04
367	Heat shock protein HSP 90-beta	4.97	P08238	513	2983.34	2054.08	0.69	4.74E-02

### Ontological analysis of differentially expressed host proteins after DENV 2 infection

**Fig 2:**
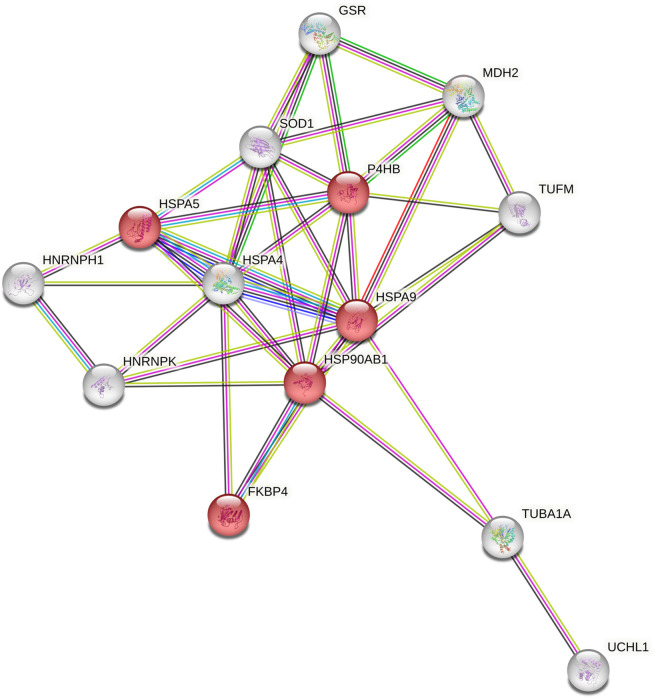
STRING analysis of 14 differentially expressed proteins after DENV 2 infection for 48 hours. Proteins identified as chaperone proteins are represented in red.

STRING analysis [19] revealed 10 biological process pathways (Supplemental Table S3 in S1File) including protein folding (5 proteins; false discovery rate 3.90E-03), chaperone-mediated protein folding (3 proteins; false discovery rate 3.30E-02) and regulation of cellular response to stress (6 proteins; false discovery rate 3.30E-02), see Fig 2.

Ontological analysis using the DAVID bioinformatics resources [20] identified 7 functional annotation clusters with enrichment score ranging from 2.93 to 1. Cluster 1 (enrichment score 2.93) contained terms relating to chaperone and protein processing in the endoplasmic reticulum, Cluster 2 (enrichment score 2.84) contained terms relating to protein folding, ATP activity and lipid and atherosclerosis, while other clusters contained terms relating to the mitochondrion and the nucleus. The full list of clusters is shown in Supplemental Table S4 in S1File. Consistently, both the STRING and DAVID analyses indicated that differentially expressed host proteins after DENV 2 infection mostly involved chaperone proteins and mitochondrion associated proteins.

### Validation of differentially expressed host protein after DENV 2 infection from 2D-gels

**Fig 3:**
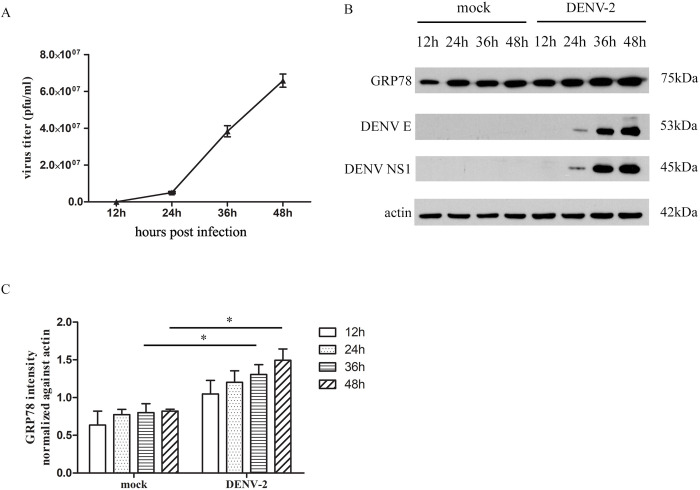
Validation of differentially expressed host protein after DENV 2 infection. DENV 2 viral titer increased in time-dependent manner (A) which corresponds to GRP78 expression in Huh-7 cells (B, C). All experiments were undertaken as independent biological triplicates. Error bars represent S.E.M. p value* < 0.05. Composite images are shown (Panel B) consisting of successive antibody probings of the same membrane which are separated by white bars. Full, uncropped western blots can be found in the supplemental materials.

To validate the 2D-gels results, Huh-7 cells were mock infected or infected with DENV 2 at MOI 5 for 12, 24, 36 and 48 hours. The supernatants were collected, and plaque assay was performed. In parallel, the cells were collected and proteins were extracted prior to western blot analysis of DENV E and NS1 proteins, and one protein identified as differentially expressed from 2D-gels, namely glucose regulated protein 78 (GRP78). The results showed that new virions released from Huh-7 cells were detectable at 24 h post-infection (Fig 3A), and this was consistent with the appearance of faint bands for E and NS1 in the western blot. Robust expression of E and NS1 proteins was observed at 36 and 48h post infection, and this coincided with significant increases in GRP78 expression as compared to mock as detected in the western blot analysis (Fig 3B and 3C). Successive probings of the same membranes were undertaken in the order shown (GRP78, followed by DENV E, NS1 and actin).

## Discussion

The liver is a well characterized site for DENV replication, and multiple lines of evidence have been generated in support of this, including disorder of the liver enzymes ALT and AST in DENV infected patients [21], detection of infected liver cells in autopsies of fatal cases [22], and the susceptibility of human primary hepatocytes to DENV infection [12]. In hepatocytes, several dengue virus receptors have been proposed such as heparan sulfate [23], glucose-regulated protein 78 [24] and the 37/67-kDa high-affinity laminin receptor [25] which makes liver cells a suitable model for dengue infection studies. Different hepatic cell lines have been shown to be susceptible to dengue infection [26]. A previous study in Huh-7 cells identified 155 differentially expressed proteins of which 64 were up-regulated and 91 were down-regulated at 24 hours post infection [5]. The probable cause for the large difference in the number of differentially expressed protein lies in the methodology. While our study used a relatively robust and comparatively insensitive methodology (2-D gel electrophoresis), the study of Pando Robles and colleagues utilized label free LC-MS [5]. However, and importantly, Pando-Robles and colleagues did not validate any of the results with a confirmatory western blot analysis [5]. However, overall Pando-Robles and colleagues found that differentially expressed proteins included those in glycolysis and gluconeogenesis [5]. In contrast our study found chaperone proteins to be the main class of differentially regulated proteins. This could possibly result from differences in timing, as our study was undertaken at 48 hours post infection, while the Pando-Robles study was undertaken at 24 hours post-infection [5].

However, of the 155 proteins identified by Pando-Robles and colleagues three (mitochondrial stress-70 protein, GRP78 and tubulin alpha-1A chain) were also identified by this study. Previous comparative proteome analysis has shown a very low concordance between studies in the proteins identified [27], and as such there is a high certainty that the proteins identified by both studies are indeed modulated by DENV infection. We appreciate that we have only validated expression of one protein (GRP78) and have not undertaken functional analysis of any of them. However, there is ample evidence already of the roles played by many of the proteins detected by this study.

Of the 14 proteins identified as differentially regulated in this study, 11 have previously been shown to be involved in DENV infection. Mitochondrial elongation factor Tu was identified as differentially regulated in a proteomic analysis of DENV infected HepG2 cells [28], while Diwakar and colleagues presented evidence that NS1 interacts with heterogeneous nuclear ribonucleoprotein H in human monocytic cells [29]. Brunetti and colleagues showed that heterogeneous nuclear ribonucleoprotein K is required for both DENV and Junin virus replication [30]. The involvement of Hsp70 in DENV infection was reported by Taguwa and colleagues [31], while Hsp90 has been shown to be able to interact with multiple DENV proteins [32] and to act as a DENV receptor protein together Hsp70 in certain cell types [33]. GRP78 is a chaperone protein predominantly found in the endoplasmic reticulum (ER). Protein disulfide isomerase (PDI) is also an enzyme located in the ER, and a study has shown that a PDI inhibitor suppresses DENV replication during antibody dependent enhancement of DENV infection in human monocytic cells [34]. An earlier study has shown that DENV NS3 gets imported to mitochondrial, where it cleaves GrpEL1, a co-chaperone of mitochondrial stress protein 70 [35]. Ubiquitin carboxyl-terminal hydrolase isozyme L1 was recently shown as a protein that was discordantly regulated by two different DENV serotypes [16]. Superoxide dismutase levels were found to be significantly elevated in children with dengue fever [36]. For the remaining three proteins (mitochondrial malate dehydrogenase, peptidyl-prolyl cis-trans isomerase FKBP4 and mitochondrial glutathione reductase) there is no prior record of a direct role of these proteins in DENV infection. However, indirect evidence is that DENV NS3 inhibits malate/pyruvate oxidation in mitochondria, resulting in decreased cellular respiration [37] suggesting that mitochondrial malate dehydrogenase may play a role in DENV infection. Similarly, studies have shown that glutathione has an inhibitory effect on DENV production [38], again indirectly implicating mitochondrial glutathione reductase as a protein with effects on DENV replication. For the last protein peptidyl-prolyl cis-trans isomerase (FKBP4) no reports were found of this proteins involvement in DENV (or other virus) infection, although this protein has been linked to glycolysis [39], which is known to be altered in DENV infected liver cells [17].

The protein validated in this study, GRP78, while primarily recognized as a chaperone protein is involved in numerous other cellular processes. In particular, GRP78 is the central regulator of the unfolded protein response (UPR), whose primary function is to adapt to cellular stresses and restore normal endoplasmic reticulum function [40,41]. GRP78 regulates the induction of the UPR by binding to three proteins in the ER lumen [40] namely Inositol-requiring protein 1 (IRE1), activating transcription factor 6 (ATF6) and protein kinase RNA-like endoplasmic reticulum kinase (PERK). Upon ER stress (such as an influx of unfolded proteins), GRP78 releases IRE1 and PERK leading to activation of both proteins through homodimerization and autophosphorylation. IRE1 and PERK leading to homodimerization and autophosphorylation and subsequent activation of each protein. Activated IRE1 excises a 26-nucleotide intron from the Xbox binding protein (XBP-1) transcript producing a transcription factor that induces the expression of ER resident chaperones [42]. Activated PERK blocks the translation of most cytoplasmic mRNAs through phosphorylation of eukaryotic initiation factor 2α (eIF-2α) and activates further downstream genes which are primarily involved in the regulation of apoptosis [43]. ATF6 becomes activated after cleavage in the Golgi compartment leading to increased expression of further chaperones [40]. Induction of the UPR as a consequence of DENV infection in several cell lines has been well documented [44–48]. The role of GRP78 has been directly examined in a number of studies. Diwaker and colleagues showed that a GRP78 inhibitor decreased DENV E protein expression in infected K562 cells [49], while Limjindaporn and colleagues showed that GRP78 knockdown reduced DENV production [50]. Wati and colleagues showed that GRP78 cleavage with toxin reduced viral proteins and viral production [51], while Songprakhon and colleagues showed that GRP78 knockdown by siRNA reduced DENV NS1 production and secretion [52]. A number of studies have shown GRP78 interacts with DENV viral proteins, including Linmjindaporn who showed an interaction between GRP78 and DENV E protein through a yeast-2-hybrid methodology and Co-immunoprecipitation [50]. Similarly, Jitobaom and colleagues showed that GRP78 interacted with both DENV E protein and VDAC [53]. Songprakhon and colleagues showed that DENV NS1 interacted with the substrate binding domain of GRP78 [52]. One study has demonstrated that GRP78 exists on the cell surface, where it can act as a receptor protein for DENV [24].

While this study focused on DENV infection, interactions between other Orthoflaviviral proteins have been shown including between GRP78 and ZIKV E protein [54,55], between GRP78 ZIKV E and NS1 proteins [56] and between GRP78 and Japanese encephalitis E virus (JEV) E protein [57]. In addition, studies have shown that GRP78 can act as a receptor protein for both ZIKV [54] and JEV [57]. Thus, our study further emphasizes the significant role of GRP78 in orthoflaviviral infections.

## Supporting information

S1 File Supplemental figures and table and full, uncropped Western blots. (PDF)

## References

[1] Postler TS, Beer M, Blitvich BJ, Bukh J, de Lamballerie X, Drexler JF, et al. Renaming of the genus Flavivirus to Orthoflavivirus and extension of binomial species names within the family Flaviviridae. Arch Virol. 2023;168(9):224. doi: 10.1007/s00705-023-05835-1 37561168

[2] Bhatt S, Gething PW, Brady OJ, Messina JP, Farlow AW, Moyes CL, et al. The global distribution and burden of dengue. Nature. 2013;496(7446):504–7. doi: 10.1038/nature12060 23563266 PMC3651993

[3] Roy SK, Bhattacharjee S. Dengue virus: epidemiology, biology, and disease aetiology. Can J Microbiol. 2021;67(10):687–702. doi: 10.1139/cjm-2020-0572 34171205

[4] Gubler DJ. Dengue and dengue hemorrhagic fever. Clin Microbiol Rev. 1998;11(3):480–96. doi: 10.1128/CMR.11.3.480 9665979 PMC88892

[5] Pando-Robles V, Oses-Prieto JA, Rodríguez-Gandarilla M, Meneses-Romero E, Burlingame AL, Batista CVF. Quantitative proteomic analysis of Huh-7 cells infected with Dengue virus by label-free LC-MS. J Proteomics. 2014;111:16–29. doi: 10.1016/j.jprot.2014.06.029 25009145

[6] Behnam MAM, Nitsche C, Boldescu V, Klein CD. The medicinal chemistry of dengue virus. J Med Chem. 2016;59(12):5622–49. doi: 10.1021/acs.jmedchem.5b01653 26771861

[7] Acosta EG, Kumar A, Bartenschlager R. Revisiting dengue virus-host cell interaction: new insights into molecular and cellular virology. Adv Virus Res. 2014;88:1–109. doi: 10.1016/B978-0-12-800098-4.00001-5 24373310

[8] Samanta J, Sharma V. Dengue and its effects on liver. World J Clin Cases. 2015;3(2):125–31. doi: 10.12998/wjcc.v3.i2.125 25685758 PMC4317605

[9] Kuo CH, Tai DI, Chang-Chien CS, Lan CK, Chiou SS, Liaw YF. Liver biochemical tests and dengue fever. Am J Trop Med Hyg. 1992;47(3):265–70. doi: 10.4269/ajtmh.1992.47.265 1355950

[10] Dissanayake HA, Seneviratne SL. Liver involvement in dengue viral infections. Rev Med Virol. 2018;28(2):10.1002/rmv.1971. doi: 10.1002/rmv.1971 29465794

[11] Win MM, Charngkaew K, Punyadee N, Aye KS, Win N, Chaisri U, et al. Ultrastructural features of human liver specimens from patients who died of dengue hemorrhagic fever. Trop Med Infect Dis. 2019;4(2):63. doi: 10.3390/tropicalmed4020063 31013708 PMC6631216

[12] Suksanpaisan L, Cabrera-Hernandez A, Smith DR. Infection of human primary hepatocytes with dengue virus serotype 2. J Med Virol. 2007;79(3):300–7. doi: 10.1002/jmv.20798 17245728

[13] Nakabayashi H, Taketa K, Miyano K, Yamane T, Sato J. Growth of human hepatoma cells lines with differentiated functions in chemically defined medium. Cancer Res. 1982;42(9):3858–63. 6286115

[14] Sithisarn P, Suksanpaisan L, Thepparit C, Smith DR. Behavior of the dengue virus in solution. J Med Virol. 2003;71(4):532–9. doi: 10.1002/jmv.10520 14556266

[15] Bradford MM. A rapid and sensitive method for the quantitation of microgram quantities of protein utilizing the principle of protein-dye binding. Anal Biochem. 1976;72:248–54. doi: 10.1016/0003-2697(76)90527-3 942051

[16] Chumchanchira C, Ramphan S, Paemanee A, Roytrakul S, Lithanatudom P, Smith DR. A 2D-proteomic analysis identifies proteins differentially regulated by two different dengue virus serotypes. Sci Rep. 2024;14(1):8287. doi: 10.1038/s41598-024-57930-1 38594317 PMC11003990

[17] Chumchanchira C, Ramphan S, Sornjai W, Roytrakul S, Lithanatudom P, Smith DR. Glycolysis is reduced in dengue virus 2 infected liver cells. Sci Rep. 2024;14(1):8355. doi: 10.1038/s41598-024-58834-w 38594438 PMC11004007

[18] Paemanee A, Hitakarun A, Wintachai P, Roytrakul S, Smith DR. A proteomic analysis of the anti-dengue virus activity of andrographolide. Biomed Pharmacother. 2019;109:322–32. doi: 10.1016/j.biopha.2018.10.054 30396090

[19] Szklarczyk D, Gable AL, Lyon D, Junge A, Wyder S, Huerta-Cepas J, et al. STRING v11: protein-protein association networks with increased coverage, supporting functional discovery in genome-wide experimental datasets. Nucleic Acids Res. 2019;47(D1):D607–13. doi: 10.1093/nar/gky1131 30476243 PMC6323986

[20] Dennis GJr, ShermanBT, HosackDA, YangJ, GaoW, LaneHC, et al. DAVID: Database for annotation, visualization, and integrated discovery. Genome Biol. 2003;4(5):P3. 12734009

[21] Kalayanarooj S, Vaughn DW, Nimmannitya S, Green S, Suntayakorn S, Kunentrasai N, et al. Early clinical and laboratory indicators of acute dengue illness. J Infect Dis. 1997;176(2):313–21. doi: 10.1086/514047 9237695

[22] Huerre MR, Lan NT, Marianneau P, Hue NB, Khun H, Hung NT, et al. Liver histopathology and biological correlates in five cases of fatal dengue fever in Vietnamese children. Virchows Arch. 2001;438(2):107–15. doi: 10.1007/s004280000329 11253111

[23] Chen Y, Maguire T, Hileman RE, Fromm JR, Esko JD, Linhardt RJ, et al. Dengue virus infectivity depends on envelope protein binding to target cell heparan sulfate. Nat Med. 1997;3(8):866–71. doi: 10.1038/nm0897-866 9256277

[24] Jindadamrongwech S, Thepparit C, Smith DR. Identification of GRP 78 (BiP) as a liver cell expressed receptor element for dengue virus serotype 2. Arch Virol. 2004;149(5):915–27. doi: 10.1007/s00705-003-0263-x 15098107

[25] Thepparit C, Smith DR. Serotype-specific entry of dengue virus into liver cells: identification of the 37-kilodalton/67-kilodalton high-affinity laminin receptor as a dengue virus serotype 1 receptor. J Virol. 2004;78(22):12647–56. doi: 10.1128/JVI.78.22.12647-12656.2004 15507651 PMC525075

[26] Lin YL, Liu CC, Lei HY, Yeh TM, Lin YS, Chen RM, et al. Infection of five human liver cell lines by dengue-2 virus. J Med Virol. 2000;60(4):425–31. doi: 10.1002/(sici)1096-9071(200004)60:4<425::aid-jmv10>3.0.co;2-a 10686026

[27] Smith DR. Global protein profiling studies of chikungunya virus infection identify different proteins but common biological processes. Rev Med Virol. 2015;25(1):3–18. doi: 10.1002/rmv.1802 25066270

[28] Pattanakitsakul S-N, Rungrojcharoenkit K, Kanlaya R, Sinchaikul S, Noisakran S, Chen S-T, et al. Proteomic analysis of host responses in HepG2 cells during dengue virus infection. J Proteome Res. 2007;6(12):4592–600. doi: 10.1021/pr070366b 17979228

[29] Diwaker D, Mishra KP, Ganju L, Singh SB. Dengue virus non-structural 1 protein interacts with heterogeneous nuclear ribonucleoprotein H in human monocytic cells. Asian Pac J Trop Med. 2016;9(2):112–8. doi: 10.1016/j.apjtm.2016.01.015 26919938

[30] Brunetti JE, Scolaro LA, Castilla V. The heterogeneous nuclear ribonucleoprotein K (hnRNP K) is a host factor required for dengue virus and Junín virus multiplication. Virus Res. 2015;203:84–91. doi: 10.1016/j.virusres.2015.04.001 25865411

[31] Taguwa S, Maringer K, Li X, Bernal-Rubio D, Rauch JN, Gestwicki JE, et al. Defining Hsp70 subnetworks in dengue virus replication reveals key vulnerability in flavivirus infection. Cell. 2015;163(5):1108–23. doi: 10.1016/j.cell.2015.10.046 26582131 PMC4869517

[32] Srisutthisamphan K, Jirakanwisal K, Ramphan S, Tongluan N, Kuadkitkan A, Smith DR. Hsp90 interacts with multiple dengue virus 2 proteins. Sci Rep. 2018;8(1):4308. doi: 10.1038/s41598-018-22639-5 29523827 PMC5844963

[33] Reyes-Del Valle J, Chávez-Salinas S, Medina F, Del Angel RM. Heat shock protein 90 and heat shock protein 70 are components of dengue virus receptor complex in human cells. J Virol. 2005;79(8):4557–67. doi: 10.1128/JVI.79.8.4557-4567.2005 15795242 PMC1069525

[34] Rawarak N, Suttitheptumrong A, Reamtong O, Boonnak K, Pattanakitsakul S-N. Protein disulfide isomerase inhibitor suppresses viral replication and production during antibody-dependent enhancement of dengue virus infection in human monocytic cells. Viruses. 2019;11(2):155. doi: 10.3390/v11020155 30781856 PMC6410196

[35] Gandikota C, Mohammed F, Gandhi L, Maisnam D, Mattam U, Rathore D, et al. Mitochondrial import of dengue virus NS3 protease and cleavage of GrpEL1, a cochaperone of mitochondrial Hsp70. J Virol. 2020;94(17):e01178-20. doi: 10.1128/JVI.01178-20 32581108 PMC7431801

[36] Ray G, Kumar V, Kapoor AK, Dutta AK, Batra S. Status of antioxidants and other biochemical abnormalities in children with dengue fever. J Trop Pediatr. 1999;45(1):4–7. doi: 10.1093/tropej/45.1.4 10191585

[37] Sousa BG, Mebus-Antunes NC, Fernandes-Siqueira LO, Caruso MB, Saraiva GN, Carvalho CF, et al. Dengue virus non-structural protein 3 inhibits mitochondrial respiration by impairing complex I function. mSphere. 2024;9(7):e0040624. doi: 10.1128/msphere.00406-24 38980068 PMC11288018

[38] Tian Y, Jiang W, Gao N, Zhang J, Chen W, Fan D, et al. Inhibitory effects of glutathione on dengue virus production. Biochem Biophys Res Commun. 2010;397(3):420–4. doi: 10.1016/j.bbrc.2010.05.108 20510876

[39] Zeng Z, Xu S, Wang R, Han X. FKBP4 promotes glycolysis and hepatocellular carcinoma progression via p53/HK2 axis. Sci Rep. 2024;14(1):26893. doi: 10.1038/s41598-024-78383-6 39505995 PMC11542027

[40] Rutkowski DT, Kaufman RJ. A trip to the ER: coping with stress. Trends Cell Biol. 2004;14(1):20–8. doi: 10.1016/j.tcb.2003.11.001 14729177

[41] Xu C, Bailly-Maitre B, Reed JC. Endoplasmic reticulum stress: cell life and death decisions. J Clin Invest. 2005;115(10):2656–64. doi: 10.1172/JCI26373 16200199 PMC1236697

[42] Lee K, Tirasophon W, Shen X, Michalak M, Prywes R, Okada T, et al. IRE1-mediated unconventional mRNA splicing and S2P-mediated ATF6 cleavage merge to regulate XBP1 in signaling the unfolded protein response. Genes Dev. 2002;16(4):452–66. doi: 10.1101/gad.964702 11850408 PMC155339

[43] Liu CY, Schröder M, Kaufman RJ. Ligand-independent dimerization activates the stress response kinases IRE1 and PERK in the lumen of the endoplasmic reticulum. J Biol Chem. 2000;275(32):24881–5. doi: 10.1074/jbc.M004454200 10835430

[44] Klomporn P, Panyasrivanit M, Wikan N, Smith DR. Dengue infection of monocytic cells activates ER stress pathways, but apoptosis is induced through both extrinsic and intrinsic pathways. Virology. 2011;409(2):189–97. doi: 10.1016/j.virol.2010.10.010 21047664

[45] Paradkar PN, Ooi EE, Hanson BJ, Gubler DJ, Vasudevan SG. Unfolded protein response (UPR) gene expression during antibody-dependent enhanced infection of cultured monocytes correlates with dengue disease severity. Biosci Rep. 2011;31(3):221–30. doi: 10.1042/BSR20100078 20858223

[46] Thepparit C, Khakpoor A, Khongwichit S, Wikan N, Fongsaran C, Chingsuwanrote P, et al. Dengue 2 infection of HepG2 liver cells results in endoplasmic reticulum stress and induction of multiple pathways of cell death. BMC Res Notes. 2013;6:372. doi: 10.1186/1756-0500-6-372 24034452 PMC3847886

[47] Umareddy I, Pluquet O, Wang QY, Vasudevan SG, Chevet E, Gu F. Dengue virus serotype infection specifies the activation of the unfolded protein response. Virol J. 2007;4:91. doi: 10.1186/1743-422X-4-91 17888185 PMC2045667

[48] Yu C-Y, Hsu Y-W, Liao C-L, Lin Y-L. Flavivirus infection activates the XBP1 pathway of the unfolded protein response to cope with endoplasmic reticulum stress. J Virol. 2006;80(23):11868–80. doi: 10.1128/JVI.00879-06 16987981 PMC1642612

[49] Diwaker D, Mishra KP, Ganju L. Effect of modulation of unfolded protein response pathway on dengue virus infection. Acta Biochim Biophys Sin (Shanghai). 2015;47(12):960–8. doi: 10.1093/abbs/gmv108 26515795

[50] Limjindaporn T, Wongwiwat W, Noisakran S, Srisawat C, Netsawang J, Puttikhunt C, et al. Interaction of dengue virus envelope protein with endoplasmic reticulum-resident chaperones facilitates dengue virus production. Biochem Biophys Res Commun. 2009;379(2):196–200. doi: 10.1016/j.bbrc.2008.12.070 19105951

[51] Wati S, Soo M-L, Zilm P, Li P, Paton AW, Burrell CJ, et al. Dengue virus infection induces upregulation of GRP78, which acts to chaperone viral antigen production. J Virol. 2009;83(24):12871–80. doi: 10.1128/JVI.01419-09 19793816 PMC2786853

[52] Songprakhon P, Limjindaporn T, Perng GC, Puttikhunt C, Thaingtamtanha T, Dechtawewat T, et al. Human glucose-regulated protein 78 modulates intracellular production and secretion of nonstructural protein 1 of dengue virus. J Gen Virol. 2018;99(10):1391–406. doi: 10.1099/jgv.0.001134 30102148

[53] Jitobaom K, Tongluan N, Smith DR. Involvement of voltage-dependent anion channel (VDAC) in dengue infection. Sci Rep. 2016;6:35753. doi: 10.1038/srep35753 27779201 PMC5078847

[54] Khongwichit S, Sornjai W, Jitobaom K, Greenwood M, Greenwood MP, Hitakarun A, et al. A functional interaction between GRP78 and Zika virus E protein. Sci Rep. 2021;11(1):393. doi: 10.1038/s41598-020-79803-z 33432092 PMC7801745

[55] Royle J, Ramírez-Santana C, Akpunarlieva S, Donald CL, Gestuveo RJ, Anaya J-M, et al. Glucose-regulated protein 78 interacts with Zika virus envelope protein and contributes to a productive infection. Viruses. 2020;12(5):524. doi: 10.3390/v12050524 32397571 PMC7290722

[56] Sornjai W, Promma P, Priewkhiew S, Ramphan S, Jaratsittisin J, Jinagool P, et al. The interaction of GRP78 and Zika virus E and NS1 proteins occurs in a chaperone-client manner. Sci Rep. 2024;14(1):10407. doi: 10.1038/s41598-024-61195-z 38710792 PMC11074156

[57] Nain M, Mukherjee S, Karmakar SP, Paton AW, Paton JC, Abdin MZ, et al. GRP78 is an important host factor for Japanese encephalitis virus entry and replication in mammalian cells. J Virol. 2017;91(6):e02274-16. doi: 10.1128/JVI.02274-16 28053106 PMC5331813

