# Supplementary material for: Increased expression of chaperone proteins in response to DENV 2 infection of Huh-7 liver cells
Source: PLoS One. 2025 Aug 1;20(8):e0329783. doi: 10.1371/journal.pone.0329783 (PMC12316284; doi:10.1371/journal.pone.0329783)

Supplemental materials

Increased expression of chaperone proteins in response to DENV 2 infection of Huh-7 liver cells

Chanida Chumchanchira[1,2], Wannapa Sornjai[2], Sittiruk Roytrakuk[3], Pathrapol Lithanatudom[4*], Duncan R. Smith[1*]

[1]PhD Degree Program in Biology, Faculty of Science, Chiang Mai University, Chiang Mai, 50200, Thailand

[2]Institute of Molecular Biosciences, Mahidol University, Nakhon Pathom, 73170, Thailand.

[3]National Center for Genetic Engineering and Biotechnology (BIOTEC), National Science and Technology Development Agency, Pathum Thani, 12120, Thailand

[4]Department of Biology, Faculty of Science, Chiang Mai University, Chiang Mai, 50200, Thailand.

*Correspondence to: Pathrapol Lithanatudom (pathrapol_li@hotmail.com) or Duncan R. Smith (duncan_r_smith@hotmail.com).

Supplemental method details.

Two-dimensional (2D)-gel electrophoresis

Cell pellets from mock- and DENV-infected Huh-7 cells were lysed using RIPA buffer (1% NP-40, 0.5% sodium deoxycholate, 0.1% sodium dodecyl sulfate, 137 mM sodium chloride, 2.7 mM potassium chloride, 4.3 mM disodium hydrogen phosphate, 1.4 mM potassium dihydrogen phosphate) containing protein inhibitor cocktail (PIC) and proteins were precipitated overnight using acetone and methanol, after which the protein pellets were dissolved in lysis C buffer (8M urea, 2M thiourea, 4% CHAPS, 20mM DTT, 1mM PMSF, 1mM benzamide) prior to determining the protein concentration by the Bradford assay. Subsequently, 250 µg of the purified proteins were loaded onto Immobiline Drystrips (pH 3-10 NL, 7 cm) containing 2% IPG buffer (Amersham Biosciences, Chalfont, United Kingdom) and 0.5% bromophenol blue and the strips were rehydrated for 12 hrs. Proteins were subjected to isoelectric focusing in a Multipor II electrophoresis system (Amersham Biosciences,) at the following voltages 300 V for 200 Vh, 1000 V for 300 Vh, a gradient to 3000 V for 4000 Vh, 5000 V for 4500 Vh and 5000 V for 3000 Vh. After focusing, the IPG strips were reduced in equilibration buffer (50 mM Tris–HCl (pH 8.8), 6 M urea, 30% v/v glycerol, 2% SDS w/v and 1% bromophenol blue) supplemented with 100 mM DTT for 15 min and then proteins were alkylated in equilibration buffer containing 150 mM iodoacetamide (IAA) for 30 minutes. The proteins were separated in the second dimension via 12.5% SDS-PAGE. Gels were stained with Coomassie Blue G250 after which the gels were visualized under a GS-900 calibrated Densitometer (Bio-Rad Laboratories, Hercules, CA). All experiments were undertaken as three independent biological replicates. Image data were analyzed using ImageMasterTM 2D Platinum version 7.0 software

(Amersham Biosciences). Statistical analysis was performed by student's t test with a p value of less than 0.05 being considered as statistically significant.

Reproduced from:

Chumchanchira C, Ramphan S, Sornjai W, Roytrakul S, Lithanatudom P, Smith DR. Glycolysis is reduced in dengue virus 2 infected liver cells. Sci Rep 2024;14:8355. Available at https://www.nature.com/articles/s41598-024-58834-w

The text is reproduced under a Creative Commons Attribution 4.0 International License, which permits use, sharing, adaptation, distribution and reproduction in any medium or format, as long as appropriate credit is given to the original author(s) and the source, provide a link to the Creative Commons license, and indicate if changes were made.

https://creativecommons.org/licenses/by/4.0/

One change has been made to the text changing the cell line from Hep3B (original) to Huh-7 (this manuscript).

Tryptic digestion and liquid chromatography–tandem mass spectrometry (GelC–MS/MS)

Briefly, the gel plugs were dehydrated with 100% acetonitrile (ACN), reduced with 10 mM DTT in 10 mM ammonium bicarbonate at 56° C for 1 h and alkylated at room temperature for 1 h in the dark in the presence of 100 mM iodoacetamide in 10 mM ammonium bicarbonate. After alkylation, the gel pieces were dehydrated with 100% ACN for 5 min. To perform in-gel digestion of protein sample, 100 ng of trypsin (10 ng/µl trypsin in 10 mM ammonium bicarbonate) was added followed by incubation at room temperature for 5 min, and then 20 µl of 10 mM ammonium bicarbonate was added to keep the gel pieces immersed throughout the digestion. The gel pieces were incubated at 37° C for 3 h. To extract digested peptides, 30 µl of 50% ACN in 0.1% formic acid was added into the gels, and then the gel pieces were incubated at room temperature for 10 min with vigorous shaking. The extracted peptides were collected and pooled together in a new tube. The pooled-extracted peptides were dried at 40° C and kept at -80° C for further analysis. Finally, dried samples were dissolved in 0.1% formic acid for subsequent mass spectrometry analysis. MS/MS analysis of tryptic peptides was performed using a SYNAPT HDMS mass spectrometer (Waters Corp., Manchester, UK). For all measurements, the mass spectrometer was operated in the V-mode of analysis with a resolution of at least 10,000 full-width half-maximum. All analyses were performed using the positive nanoelectrospray ion mode. The time-of-flight analyzer of the mass spectrometer was externally calibrated with [Glu1]fibrinopeptide B from m/z 50 to 1600 with acquisition lock mass corrected using the monoisotopic mass of the doubly charged precursor of [Glu1]fibrinopeptide B. The reference sprayer was switched at a frequency of 20 s. Accurate mass LC–MS data were acquired with the data direct acquisition mode. The energy of the trap was set at a collision energy of 6 V. In transfer collision energy control, low energy was set at 4 V. The quadrupole mass analyzer was adjusted such that ions from m/z 300 to 1800 were efficiently transmitted. The MS/MS

survey was over the range 50 to 1990 Da and scan time was 0.5 s. For proteins quantitation, DeCyder MS Differential Analysis software (DeCyderMS, GE Healthcare) was used.

The text is reproduced under a Creative Commons Attribution BY-NC-ND 4.0, which permits copy and redistribution of the material in any medium or format. To comply with the license appropriate credit must be given to the original publication, provide a link to the license and indicate if changes were made.

1. Reproduced from Paemanee A, Hitakarun A, Wintachai P, Roytrakul S, Smith DR. A proteomic analysis of the anti-dengue virus activity of andrographolide. Biomed Pharmacother. 2019 Jan;109:322-332. doi: 10.1016/j.biopha.2018.10.054. Epub 2018 Nov 3
2. https://creativecommons.org/licenses/by-nc-nd/4.0/
3. No changes to the text have been made.

Supplemental Figure S1

Optimization of infection of Huh-7 cells with DENV

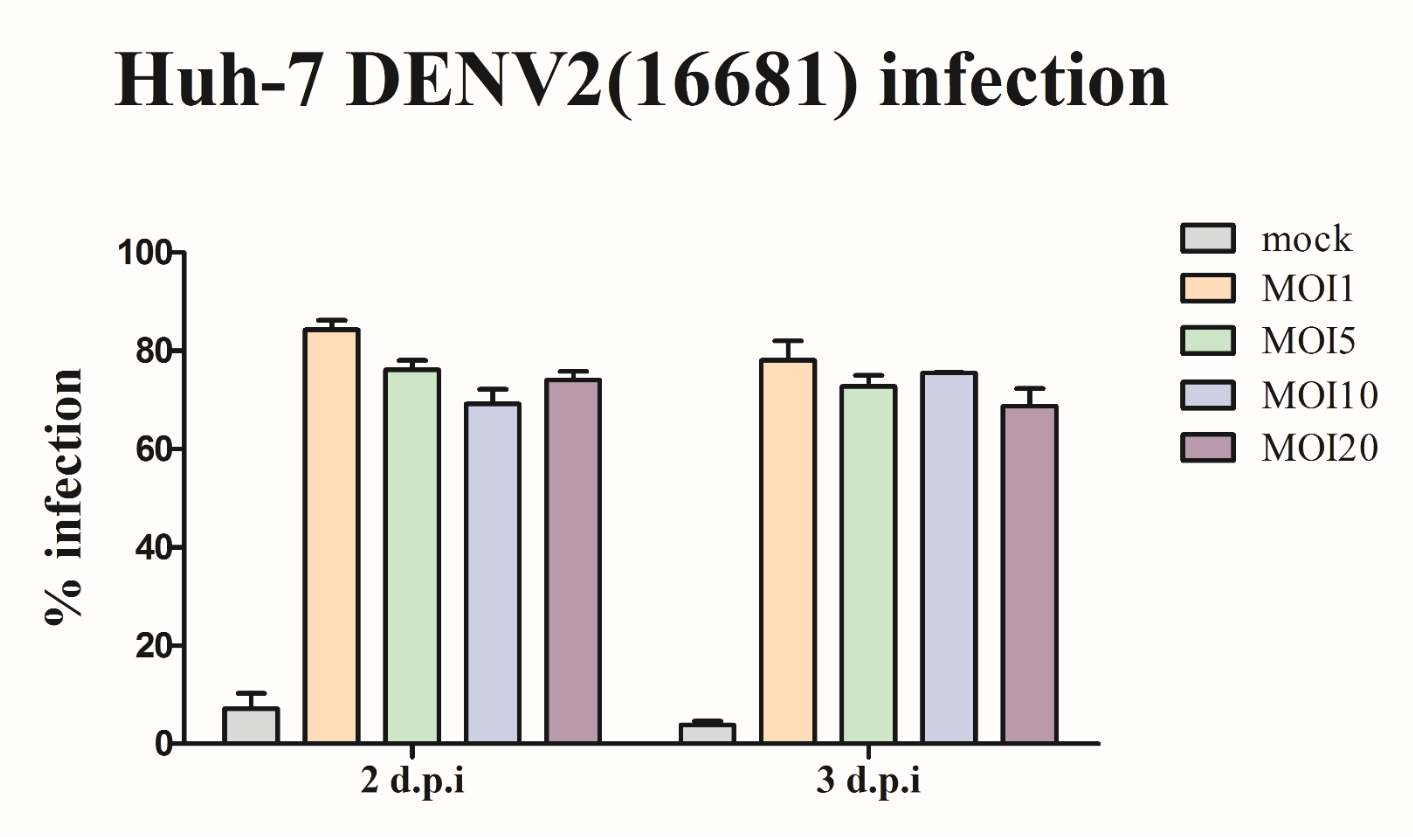

Supplemental Figure S2.
Three independent biological replicate 2-D gels of Huh-7 cells after mock infection for 48 hours.

Replicate 1

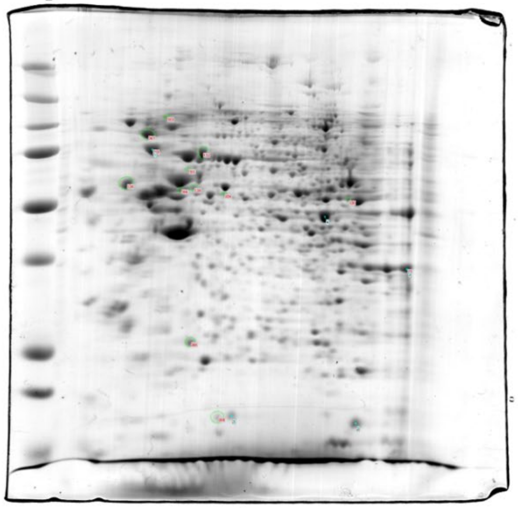

Replicate 2

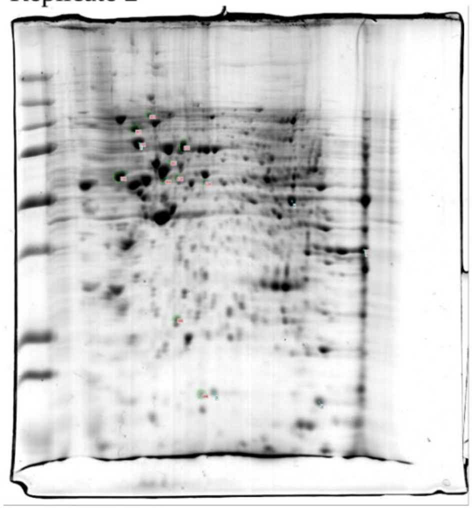

Replicate 3

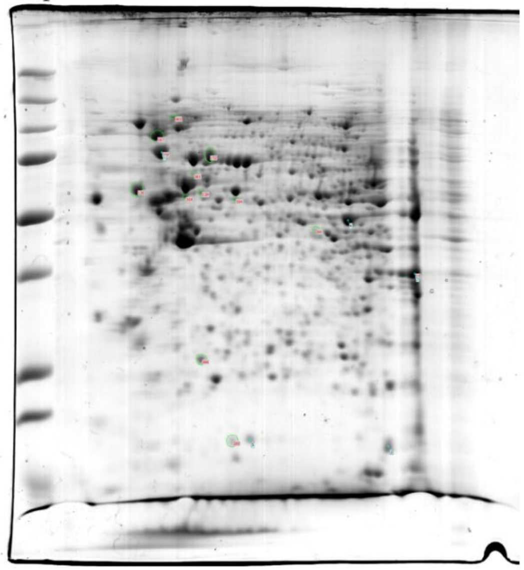

Supplementary Fig S3.

Three independent biological replicate 2-D gels of Huh-7 cells after DENV 2 infection for 48 hours.

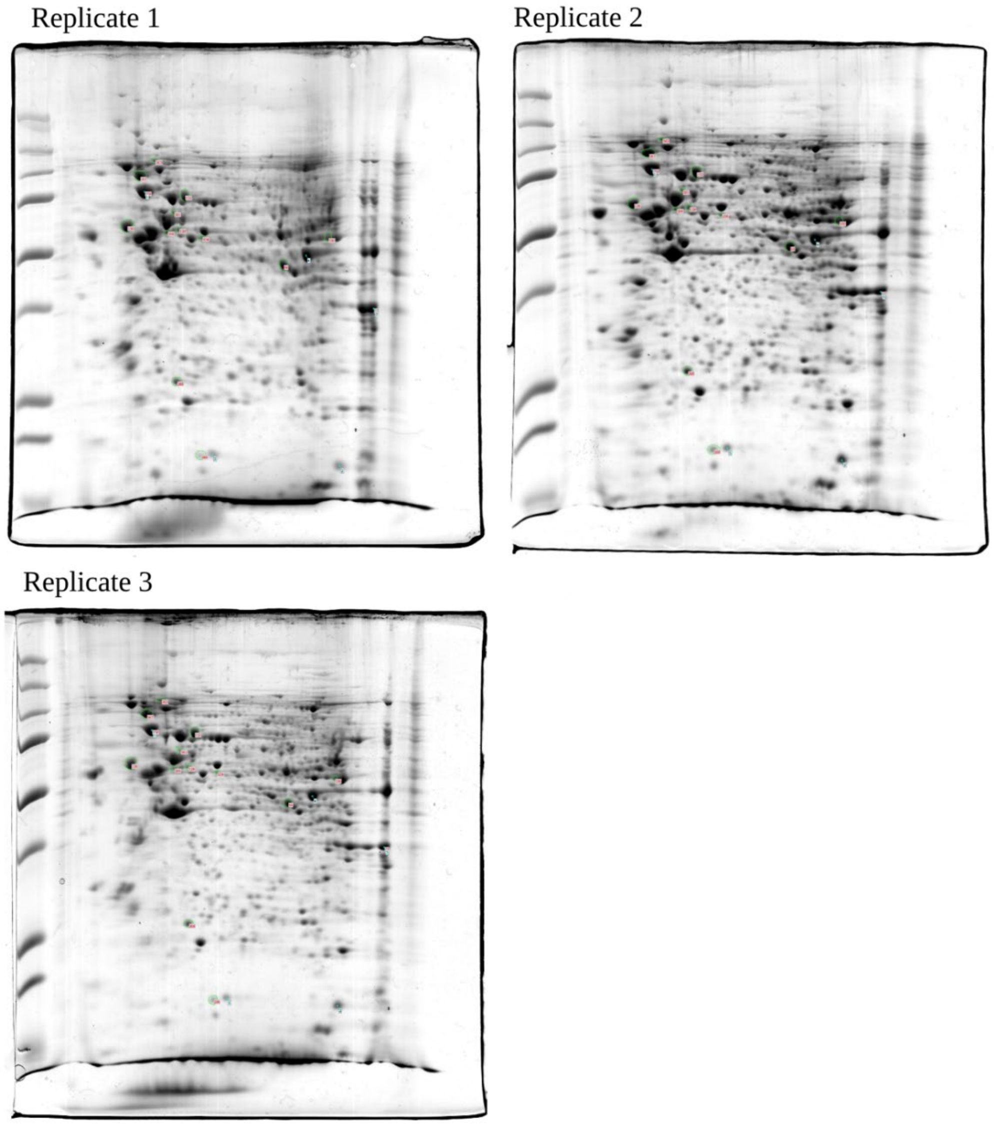

Supplemental Table S1. Antibodies and dilutions

Name of antibody	Source	Type	Dilution	Detail
Anti-GRP78 BiP (ab21685; Abcam plc, Cambridge, UK)	rabbit	polyclonal	1:8000	1°Ab
Anti-NS1 (DENV-2)(PA5-278885; Pierce, Rockford, IL, USA)	rabbit	polyclonal	1:5000	1°Ab
Pan specific anti-dengue virus type 1-4 antibody (MA1-27093; Pierce, Rockford, IL, USA)	mouse	polyclonal	1:3000	1°Ab
β-Actin (C4)-HRP (sc-4778HRP; Santa Cruz, Biotechnology Inc., Texas, USA)	mouse	monoclonal	1:50000	1°Ab
HRP-conjugated goat anti-mouse IgG (A4416; Sigma, MO, USA)	goat	polyclonal	1:5000	2°Ab
HRP-conjugated goat anti-rabbit IgG (31460; Pierce, IL, USA)	goat	polyclonal	1:5000	2°Ab

Supplemental Table S2: Identification of proteins by replicate

Identified proteins	mock infection			DENV 2 infection		
	replicate 1	replicate 2	replicate 3	replicate 1	replicate 2	replicate 3
Malate dehydrogenase, mitochondrial	✓	✓	✓	✓	✓	✓
Elongation factor Tu, mitochondrial	-	-	✓	✓	✓	✓
Heterogeneous nuclear ribonucleoprotein H	✓	✓	✓	✓	✓	✓
Peptidyl-prolyl cis-trans isomerase FKBP4	✓	✓	✓	✓	✓	✓
Protein disulfide-isomerase	✓	✓	✓	✓	✓	✓
Stress-70 protein, mitochondrial	✓	✓	✓	✓	✓	✓
Endoplasmic reticulum chaperone BiP	✓	✓	✓	✓	✓	✓
Superoxide dismutase [Cu-Zn]	✓	✓	✓	✓	✓	✓
Glutathione reductase, mitochondrial	✓	-	-	✓	✓	✓
Heterogeneous nuclear ribonucleoprotein K	✓	✓	✓	✓	✓	✓
Heat shock 70 kDa protein 4	✓	✓	✓	✓	✓	✓
Tubulin alpha-1A chain	✓	✓	✓	✓	✓	✓
Ubiquitin carboxyl-terminal hydrolase isozyme L1	✓	✓	✓	✓	✓	✓
Heat shock protein HSP 90-beta	✓	✓	✓	✓	✓	✓

Supplementary Table S3.
Functional enrichments in biological processes analyzed by the STRING bioinformatic analysis software.

**Biological Process(GO)**			
Pathway ID	*Pathway discription*	*Count**	*FDR***
GO:0006457	Protein folding	5	0.0039
GO:0006986	Response to unfolded protein	4	0.0330
GO:0035690	Cellular response to drug	3	0.0330
GO:0035722	interleukin-12-mediated signaling pathway	3	0.0330
GO:0042221	Response to chemical	11	0.0330
GO:0061077	Chaperone-mediated protein folding	3	0.0330
GO:0080135	Regulation of cellular response to stress	6	0.0330
GO:0042493	Response to drug	4	0.0422
GO:0070887	Cellular response to chemical stimulus	9	0.0422
GO:0010033	Response to organic substance	9	0.0461

*Count: Count in gene set

**FDR: False discovery rate.

Supplemental Table S4

Functional annotation clustering results from the DAVID Bioinformatics Resource. Classification stringency: Medium. The output was generated based on input of Uniprot_accessions: P40926, P49411, P31943, Q02790, P07237, P38646, P11021, P00441, P00390, P61978, P34932, Q71U36, P09936 and P08238

**Annotation Cluster 1**	**Enrichment Score: 2.93**	**Count**	**P_value**	**Benjamini**
UP_KW_MOLECULAR_FUNCTION	Chaperone	5	6.40E-05	1.00E-03
GOTERM_CC_DIRECT	macromolecular complex	5	7.50E-04	1.80E-02
GOTERM_CC_DIRECT	melanosome	3	1.90E-03	2.00E-02
KEGG_PATHWAY	Protein processing in endoplasmic reticulum	3	2.10E-02	2.50E-01

**Annotation Cluster 2**	**Enrichment Score: 2.84**	**Count**	**P_value**	**Benjamini**
GOTERM_BP_DIRECT	protein folding	5	4.10E-06	1.00E-03
GOTERM_MF_DIRECT	heat shock portein binding	4	1.20E-05	7.90E-04
INTERPRO	Heat shock protein 70 family	3	4.40E-05	1.20E-03
INTERPRO	Heat shoock protein 70, conserved site	3	4.40E-05	1.20E-03
UP_KW_MOLECULAR_FUNCTION	Chaperone	5	6.40E-05	1.00E-03

GOTERM_MF_DIRECT	protein binding involved in protein folding	3	5.00E-04	1.40E-02
GOTERM_MF_DIRECT	ubiquitin protein ligase binding	4	1.10E-03	2.20E-02
UP_KW_MOLECULAR_ FUNCTION	Stress response	3	2.60E-03	2.30E-02
GOTERM_MF_DIRECT	unfolded protein binding	3	3.50E-03	4.80E-02
KEGG_PATHWAY	Antigen processing and presentation	3	4.60E-03	1.00E-01
GOTERM_MF_DIRECT	ATP binding	5	1.70E-02	1.60E-01
GOTERM_MF_DIRECT	ATPase activity	3	2.40E-02	1.70E-01
KEGG_PATHWAY	Lipid and atherosclerosis	3	3.20E-02	2.80E-01
UP_KW_LIGAND	ATP-binding	4	2.30E-01	8.30E-01
UP_SEQ_FEATURE	COMPBIAS:Basic and acidic residues	5	5.00E-01	1.00E+00

**Annotation Cluster 3**	**Enrichment Score: 2.61**	**Count**	**P_value**	**Benjamini**
GOTERM_CC_DIRECT	mitochondrial matrix	4	1.70E-03	2.00E-02
UP_KW_CELLULAR_ COMPONENT	Mitochondrion	6	1.70E-03	2.30E-02
UP_KW_DOMAIN	transit peptide	4	2.90E-03	1.70E-02
UP_SEQ_FEATURE	TRANSIT:Mitochondrion	4	4.50E-03	4.80E-01

Annotation Cluster 4	Enrichment Score: 2.13	Count	P_value	Benjamini
UP_KW_MOLECULAR_ FUNCTION	Chaperone	5	6.40E-05	1.00E-03
GOTERM_CC_DIRECT	focal adhesion	4	2.10E-03	2.00E-02
GOTERM_MF_DIRECT	enzyme binding	3	2.80E-02	1.80E-01
UP_KW_DOMAIN	Signal	3	7.50E-01	9.70E-01

Annotation Cluster 5	Enrichment Score: 1.16	Count	P_value	Benjamini
KEGG_PATHWAY	Parkinson disease	4	4.60E-03	1.00E-01
KEGG_PATHWAY	Pathways of neurodegeneration - multiple disease	4	2.30E-02	2.50E-01
KEGG_PATHWAY	Prion disease	3	5.00E-02	3.60E-01
KEGG_PATHWAY	Amyotrophic lateral sclerosis	3	8.30E-02	5.20E-01
GOTERM_CC_DIRECT	plasma membrane	5	4.40E-01	1.00E+00
UP_KW_MOLECULAR_ FUNCTION	Hydrolase	3	5.60E-01	1.00E+00

**Annotation Cluster 6**	**Enrichment Score: 1.05**	**Count**	**P_value**	**Benjamini**
GOTERM_MF_DIRECT	identical protein binding	5	2.50E-02	1.70E-01
UP_KW_PTM	Ubl conjugation	6	7.20E-02	1.60E-01
GOTERM_CC_DIRECT	necleoplasm	6	8.60E-02	3.50E-01
UP_KW_CELLULAR_COMPONENT	Nucleus	6	4.10E-01	8.90E-01

**Annotation Cluster 7**	**Enrichment Score: 1**	**Count**	**P_value**	**Benjamini**
UP_KW_PTM	Ubi conjugation	6	7.20E-02	1.60E-01
UP_KW_PTM	Isopeptide bond	5	7.50E-02	1.60E-01
UP_SEQ_FEATURE	CROSSLNK:Glycyl lysine isopeptide (Lys-Gly) (interchain with G-Cter in SUMO2)	3	1.90E-01	1.00E+00

Uncropped western blots

Figure 3B

(GRP78)

replicate 1

replicate 2

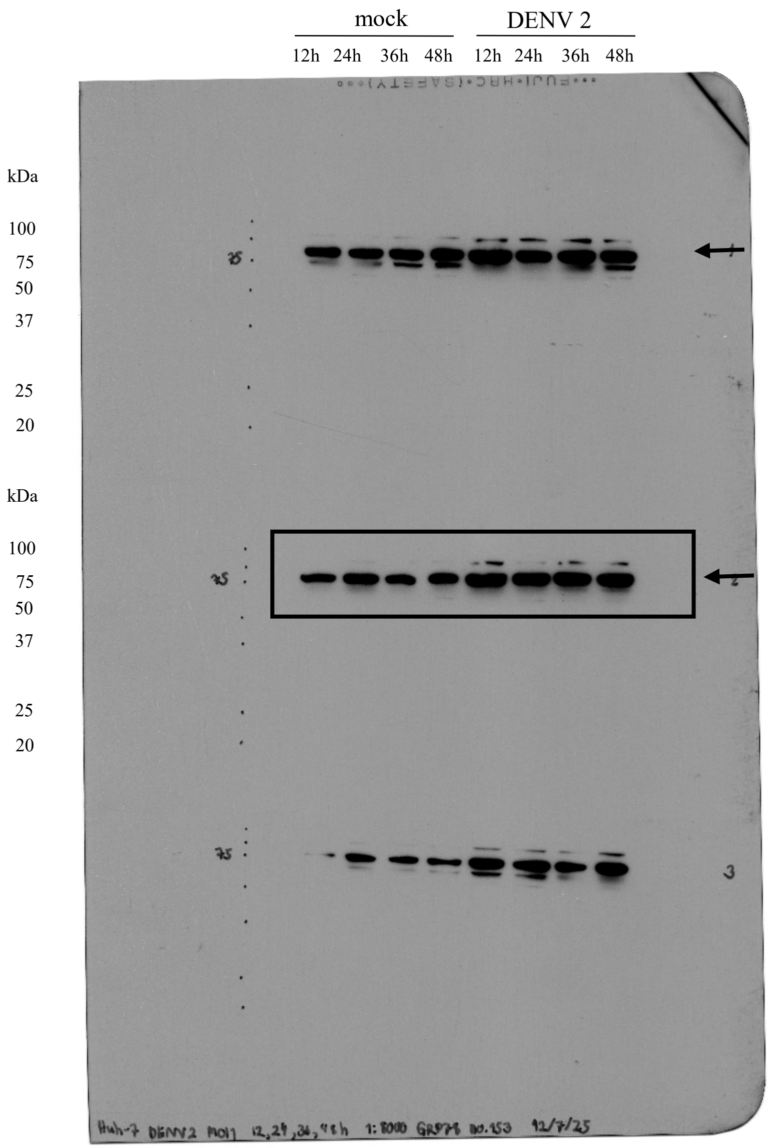

Figure 3B

(GRP78)

replicate 3

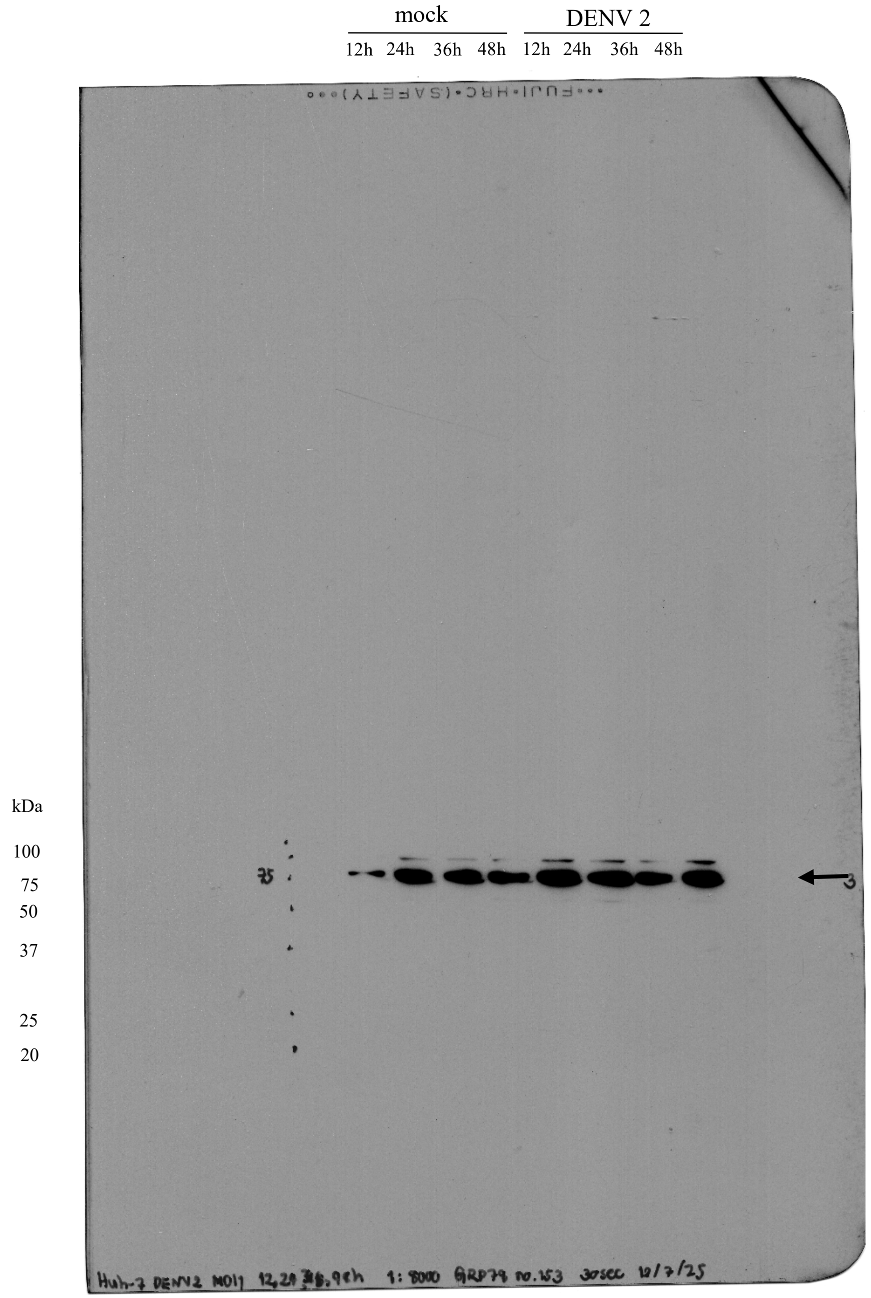

Figure 3B

(DENV E)

replicate 1

replicate 2

replicate 3

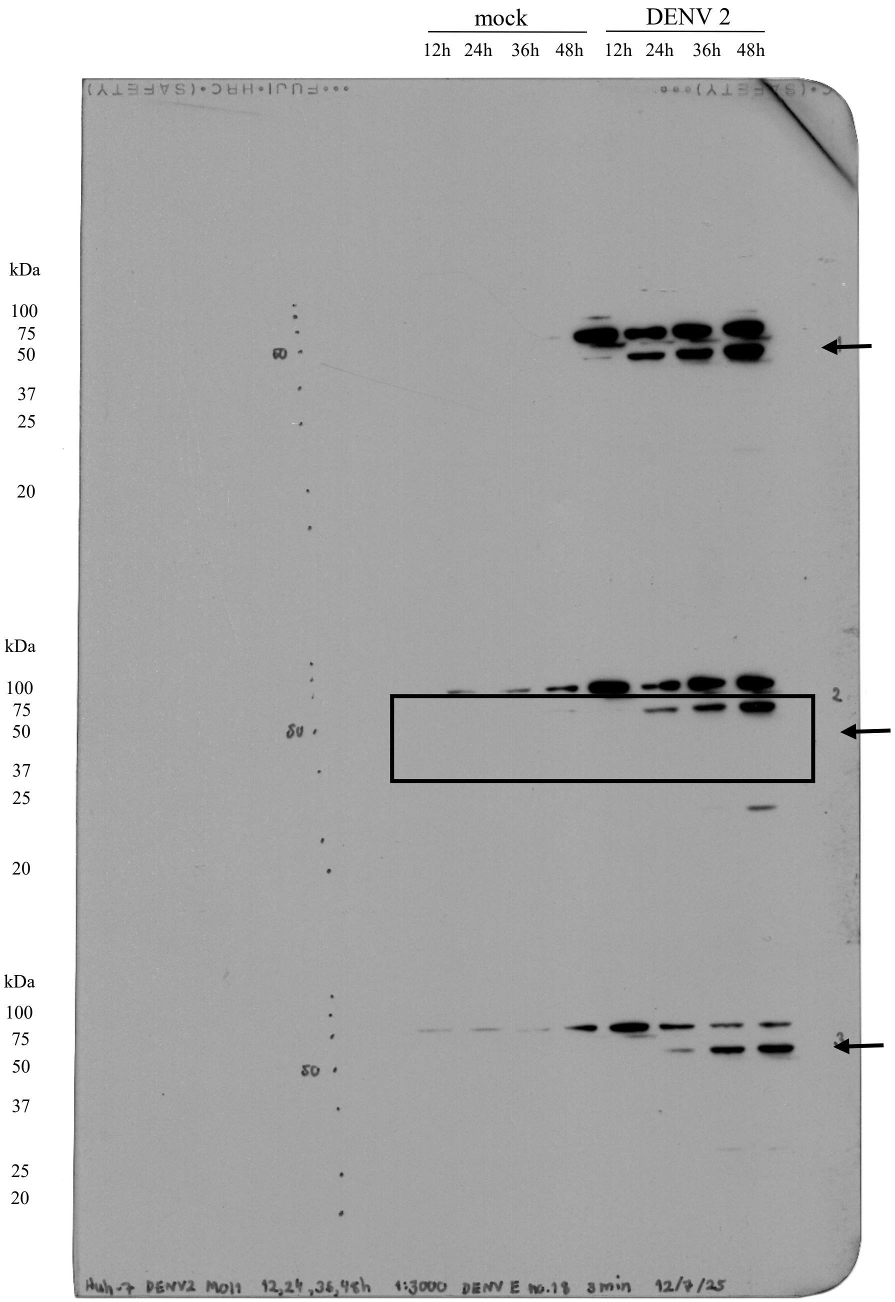

Figure 3B

(DENV NS1)

replicate 1

replicate 2

replicate 3

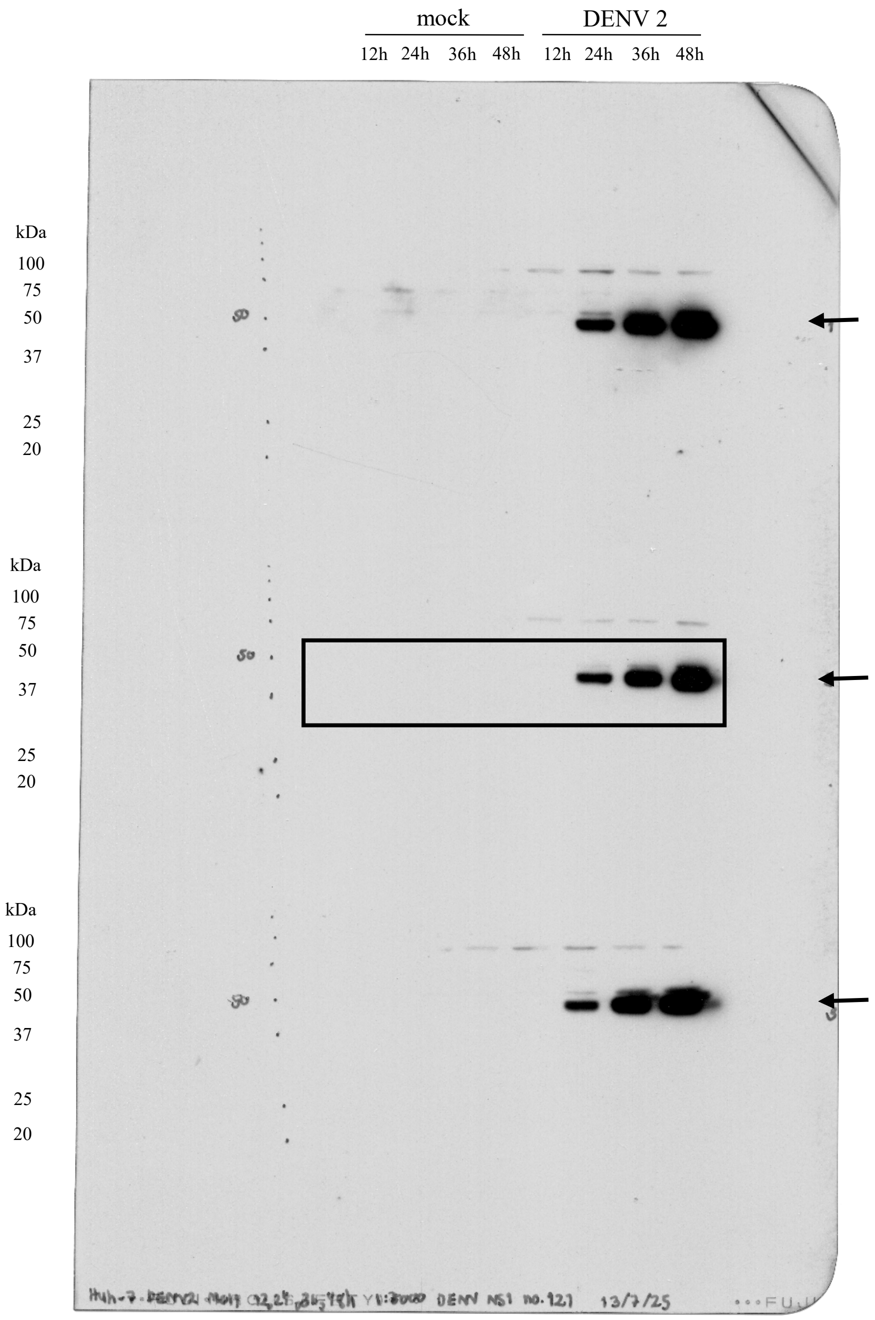

Figure 3B

(Actin)

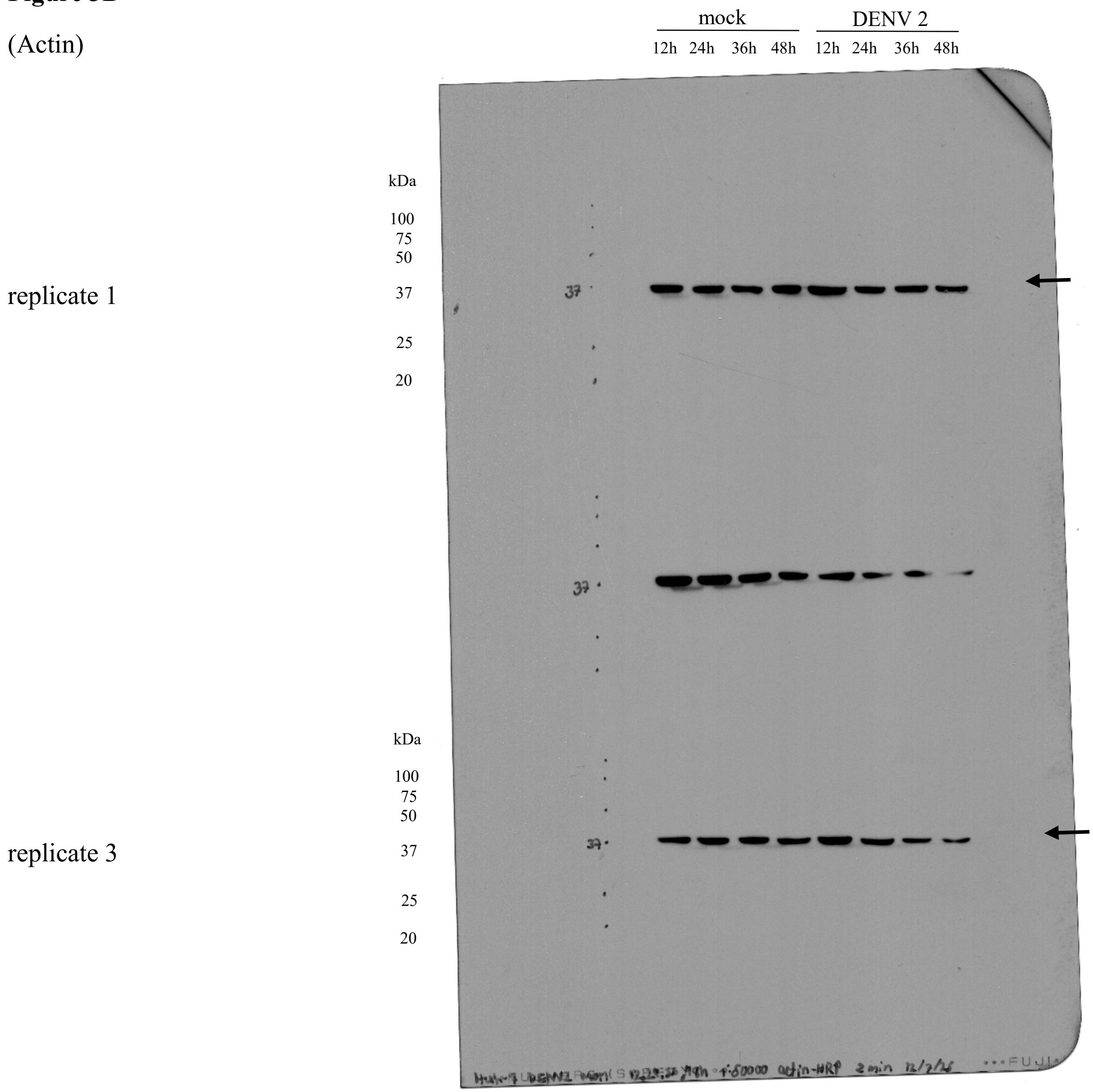

Figure 3B

(Actin)

replicate 2

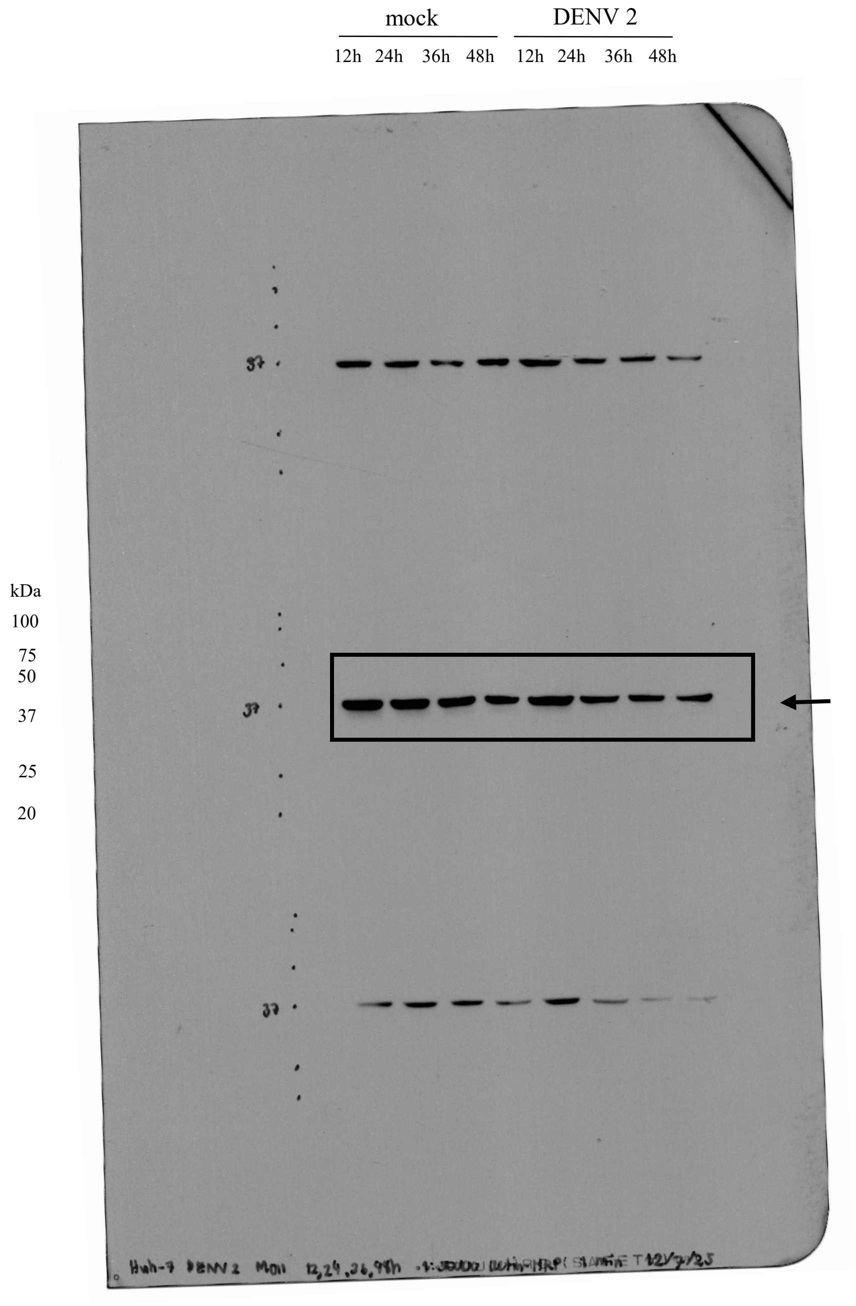

Supplement: S1 File — (PDF) [file pone.0329783.s001.pdf]
